# Enhanced Discrimination of Malignant from Benign Pancreatic Disease by Measuring the CA 19-9 Antigen on Specific Protein Carriers

**DOI:** 10.1371/journal.pone.0029180

**Published:** 2011-12-29

**Authors:** Tingting Yue, Kevin A. Maupin, Brian Fallon, Lin Li, Katie Partyka, Michelle A. Anderson, Dean E. Brenner, Karen Kaul, Herbert Zeh, A. James Moser, Diane M. Simeone, Ziding Feng, Randall E. Brand, Brian B. Haab

**Affiliations:** 1 Laboratory of Cancer Immunodiagnostics, Van Andel Institute, Grand Rapids, Michigan, United States of America; 2 Fred Hutchinson Cancer Research Center, Seattle, Washington, United States of America; 3 University of Michigan Medical Center, Ann Arbor, Michigan, United States of America; 4 Northshore University Health System, Evanston, Illinois, United States of America; 5 University of Pittsburgh Medical Center, Pittsburgh, Pennsylvania, United States of America; 6 Cell and Molecular Biology Program, Michigan State University, East Lansing, Michigan, United States of America; Kings College, London, United Kingdom

## Abstract

The CA 19-9 assay detects a carbohydrate antigen on multiple protein carriers, some of which may be preferential carriers of the antigen in cancer. We tested the hypothesis that the measurement of the CA 19-9 antigen on individual proteins could improve performance over the standard CA 19-9 assay. We used antibody arrays to measure the levels of the CA 19-9 antigen on multiple proteins in serum or plasma samples from patients with pancreatic adenocarcinoma or pancreatitis. Sample sets from three different institutions were examined, comprising 531 individual samples. The measurement of the CA 19-9 antigen on any individual protein did not improve upon the performance of the standard CA 19-9 assay (82% sensitivity at 75% specificity for early-stage cancer), owing to diversity among patients in their CA 19-9 protein carriers. However, a subset of cancer patients with no elevation in the standard CA 19-9 assay showed elevations of the CA 19-9 antigen specifically on the proteins MUC5AC or MUC16 in all sample sets. By combining measurements of the standard CA 19-9 assay with detection of CA 19-9 on MUC5AC and MUC16, the sensitivity of cancer detection was improved relative to CA 19-9 alone in each sample set, achieving 67–80% sensitivity at 98% specificity. This finding demonstrates the value of measuring glycans on specific proteins for improving biomarker performance. Diagnostic tests with improved sensitivity for detecting pancreatic cancer could have important applications for improving the treatment and management of patients suffering from this disease.

## Introduction

Several factors contribute to the extremely poor prognosis associated with pancreatic cancer, including the resistance of the disease to available therapeutic options, its tendency to metastasize at small primary tumor sizes, and its induction of cachexia [1]. The lack of effective tools for accurately detecting and diagnosing the disease at early stages further contributes to the problems in treating the disease. Because of the lack of early detection methods, most pancreatic cancers are detected at an advanced stage. Furthermore, because established disease can be difficult to diagnose due to clinical similarities with certain benign diseases such as chronic pancreatitis [2], some patients may receive sub-optimal treatment. Current diagnostic modalities include non-invasive imaging, endoscopic ultrasound, and cytology based on fine-needle aspiration [3]. These methods are useful for identifying pancreatic abnormalities and rendering an accurate diagnosis in many cases, but they come with high cost, significant expertise required for interpretation, and inherent uncertainty. Molecular markers could provide a useful complement to imaging and cytology methods, since they have the potential to provide objective information in an inexpensive, routine assay. Therefore, identifying and developing molecular markers providing useful diagnostic information for pancreatic cancer is a high priority.

The CA 19-9 serum marker is elevated in the majority of pancreatic cancer patients but does not achieve the performance required for either early detection or diagnosis, due to both false positive and false negative readings [4]. Patients with biliary obstruction, liver diseases, and pancreatitis may have elevations in CA 19-9, so its elevation is not exclusively specific for malignancy. In addition, some patients with cancer do not show elevation [5], reducing its usefulness for confirming cancer in suspect cases. The information from CA 19-9 is useful, in coordination with other clinical factors, for monitoring disease progression in patients receiving therapy [6]. Methods to improve detection of the patients who are low in CA 19-9, or to reduce false detection of patients with non-malignant elevations in CA 19-9, would be useful for developing effective pancreatic cancer biomarkers.

The nature of the CA 19-9 antigen suggests a strategy for potentially improving biomarker performance. The CA 19-9 antigen is a carbohydrate structure called sialyl LewisA (part of the Lewis family of blood group antigens) with the sequence Neu5Acα2,3Galβ1,3(Fucα1,4)GlcNAc. Sialyl LewisA is synthesized by glycosyltransferases that sequentially link the monosaccharide precursors onto both N-linked and O-linked glycans. Sialyl LewisA is not found at a high level in normal tissues, but it is found in embryonic tissue [7] and overexpressed in certain epithelial cancers and inflammatory conditions [4]. It is attached to many different proteins, including mucins, carcinoembryonic antigen [8], [9], and circulating apolipoproteins [10]. In the standard CA 19-9 clinical assay, a monoclonal antibody captures and detects the CA 19-9 antigen in a sandwich ELISA format, which measures the CA 19-9 antigen on many different carrier proteins [9].

It is possible that the carrier proteins of the CA 19-9 antigen are different between disease states, as suggested earlier [10], [11]. If that is the case, the detection of the CA 19-9 antigen on particular carrier proteins may yield improved discrimination of the disease states, in comparison to measurements of total CA 19-9. We previously demonstrated a method for detecting the level of particular glycans on individual proteins captured out of biological solutions [12], [13], [14]. Antibody arrays capture multiple, different proteins, and glycan-binding lectins or antibodies detect the glycan levels on the captured proteins. This method provides sensitive and reproducible measurements in low sample volumes and is compatible with high-throughput sample processing [15]. Previous work using this method showed that the mucins MUC1, MUC5AC, and MUC16 are major cancer-associated carriers of the CA 19-9 antigen in the blood [13]. In this work, we tested the hypothesis that the detection of the CA 19-9 antigen on specific proteins can yield improved biomarker performance over total CA 19-9 in the detection of cancer. We tested this hypothesis for the particularly difficult diagnostic problem of differentiating pancreatic cancer patients from pancreatitis patients [2], for which CA 19-9 alone does not give sufficient performance to be clinically useful. We show that clear distinctions exist between patients in the proteins that carry the CA 19-9 antigen, and that a biomarker panel based on the detection of the CA 19-9 on specific proteins accurately identifies a greater percentage of cancer patients than the conventional CA 19-9 assay.

## Results

### Profiling the CA 19-9 antigen on specific proteins

**Figure 1:**
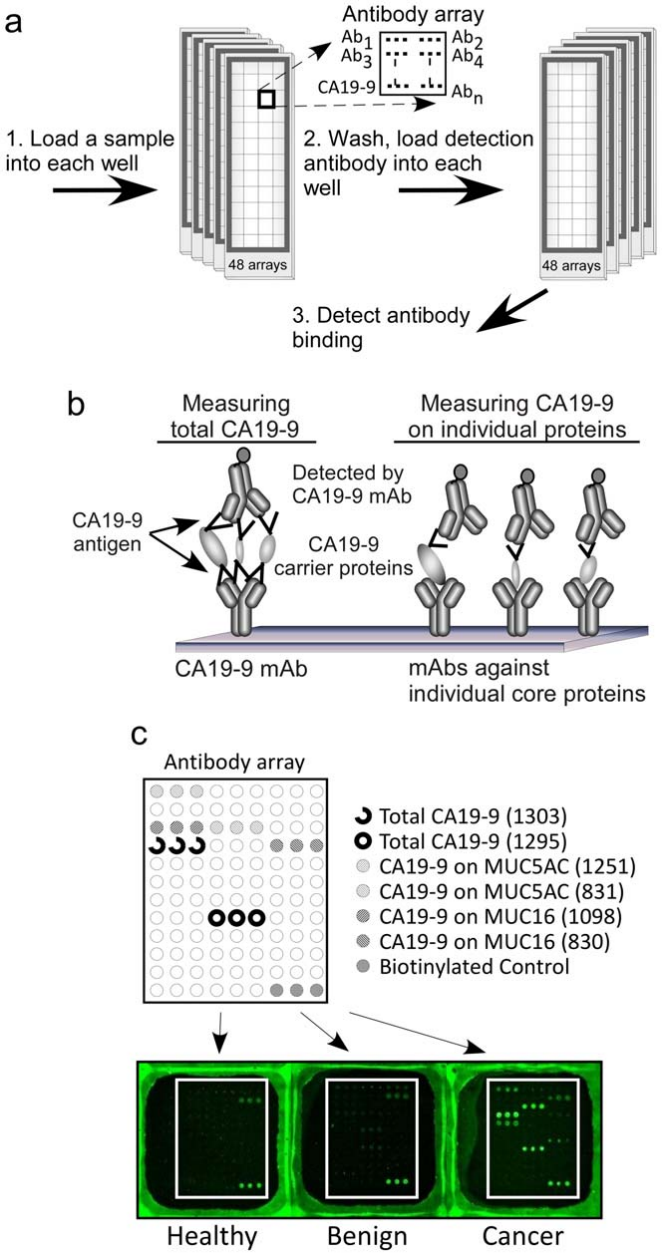
Detection of total CA19-9 and CA 19-9 on individual proteins using antibody arrays. a) High-throughput sample processing and array-based sandwich assays for CA19-9 detection. Forty-eight identical arrays are printed on one microscopic slide, segregated by hydrophobic wax boundaries (left). A set of serum or plasma samples are incubated on the arrays in random order, and the arrays for the entire sample set are probed with the CA 19-9 detection antibody (right). b) Molecular detail. Total CA19-9 is measured at the CA19-9 capture antibody (left), and CA19-9 on specific proteins is measured at the individual antibodies against those proteins (right). b) Representative raw image data from each of the sample groups. Triplicates of each antibody were randomly positioned on the array, as indicated for selected antibodies.

We used antibody arrays to measure the level of the CA 19-9 antigen on specific proteins in multiple samples. Serum and plasma samples were incubated on antibody arrays, and the arrays were probed with the CA 19-9 antibody (Fig. 1a) to detect either the total level of its target antigen (detected at the CA 19-9 capture antibody) or its level on particular proteins (detected at the capture antibodies against specific proteins) (Fig. 1b). Each antibody was printed in triplicate, and the locations of the triplicate spots were randomized to minimize potential positional bias within each array. The ability to print and process 48 antibody arrays on a single microscopic slide enabled the efficient evaluation of multiple clinical samples (Fig. 1a). Dilution curves of pooled serum/plasma samples generated in our previous study [13] confirmed the detection of the targeted proteins or glycans in the linear response range at a two-fold dilution, and the use of negative control antibodies (mouse mAbs lacking specificity for any human protein) and negative control arrays (arrays incubated with PBS buffer instead of serum or plasma) confirmed a lack of non-specific binding to the capture antibodies by the detection reagents. The various capture antibodies displayed distinct binding patterns (Fig. 1c), consistent with the unique specificities of the antibodies.

In order to determine which antibodies should be used to profile CA 19-9 levels over many patients, we profiled a pilot set of 12 serum samples (6 from pancreatic cancer patients and 6 from pancreatitis patients) using arrays containing 58 different antibodies (Fig. S1). The antibodies targeted a variety of serum proteins, mucins, matrix proteins, adhesion proteins, and cytokines (Table S1). Antibodies that were likely to capture a protein carrying the CA 19-9 antigen were identified based on signal relative to background and standard deviation across the samples. In addition, those binding potential markers of disease were identified by statistical comparison between the patient groups. Eight candidate protein carriers were identified, with four of them showing differences between the groups, and in follow up experiments using smaller arrays (16 antibodies, Table S2) and a larger sample set (20 case and 24 control samples), two protein carriers were consistently identified: MUC16 and MUC5AC. MUC1 was significant in the larger scale experiment. This result is consistent with a previous study that showed increased levels and altered glycosylation of these proteins in the blood of pancreatic cancer patients [13].

Based on the above result, subsequent experiments were performed using arrays targeting CA 19-9 and the mucin proteins MUC1, MUC5AC, and MUC16 (see Table S3 for information on the antibodies). Four to five different monoclonal antibodies were used for each protein, and each antibody was printed in triplicate. Three independent sample sets, obtained from three different institutions (Table 1), were processed, and sample set 1 was processed blinded and in triplicate on different days with distinct batches of microarrays. The third replicate of sample set 1 was primarily used for the analysis here due to minor improvements in the methods used for that replicate.

**Table 1:** Sets of serum and plasma used in the study.

Set #	Set provider	Early-stage cancer (Stage I, II)	Undetermined stage cancer	Late-stage cancer (Stage III, IV)	Pancreatitis	Healthy	Total
1	University of Pittsburgh (UP)	54	13	58	51	54	
2	Evanston Northwestern Healthcare (ENH)	60	9	63	36	52	531
3	University of Michigan (UM)		28		15	38	

**Figure 2:**
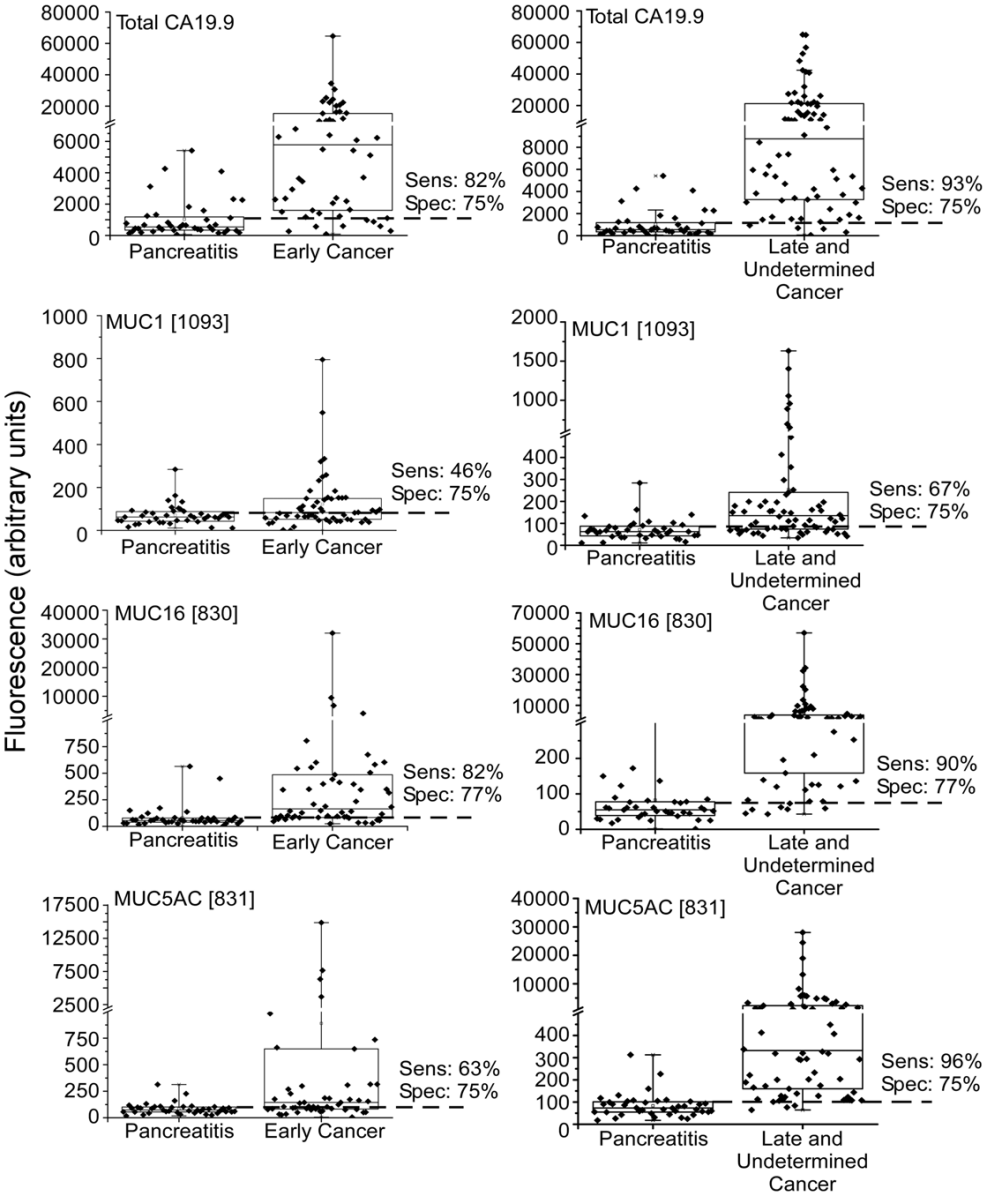
Total CA 19-9 levels and CA 19-9 on specific proteins. The fluorescence values for the total CA 19-9 (top), CA19-9 on MUC1 (second row), CA 19-9 on MUC16 (third row), and CA 19-9 on MUC5AC (fourth row) are shown for each sample group. The left column compares samples from pancreatitis patients to samples from early-stage pancreatic cancer patients, and the right column compares pancreatitis to late-stage cancer. The sensitivity and specificity at the threshold indicated by the dash line are given.

The first goal of the analysis was to determine whether the detection of the CA 19-9 antigen on any individual protein performed as well or better than the standard CA 19-9 assay (referred to as total CA 19-9). Each of the proteins MUC1, MUC5AC, and MUC16 showed significantly higher levels in the cancer patients than in the pancreatitis patients, both for early and late stage cancers (Fig. 2). (Results from the best-performing capture antibodies are shown; the other antibodies targeting these proteins showed similar results but weaker discrimination between the groups.) The detection of CA 19-9 on MUC16 had performance statistically equivalent to that of total CA 19-9, with a detection of early-stage cancer at 82% sensitivity and 77% specificity, and a detection of late-stage cancer at 90% sensitivity and 77% specificity. Sample sets 2 and 3 also showed the same relationships (not shown). (Sample set 3 showed evidence of systematic bias between the cases and controls, so was used to confirm relationships between markers but is not presented in the subsequent analyses.) Therefore, using these proteins, the detection of CA 19-9 on an individual protein does not exceed the performance of the standard CA 19-9 assay. However, the very good discrimination between groups shows that these proteins are major disease-associated carriers of the CA 19-9 antigen.

### Patient diversity in CA 19-9 carrier proteins

We next investigated the relationships between total CA 19-9 and CA 19-9 on individual proteins to determine whether elevations occur independently from one another. If non-overlapping patients are elevated in separate markers, the markers could be used together to yield improved performance. This potential was supported by the lack of significant correlation between total CA 19-9 and CA 19-9 on individual proteins or between the individual proteins (not shown).

**Figure 3:**
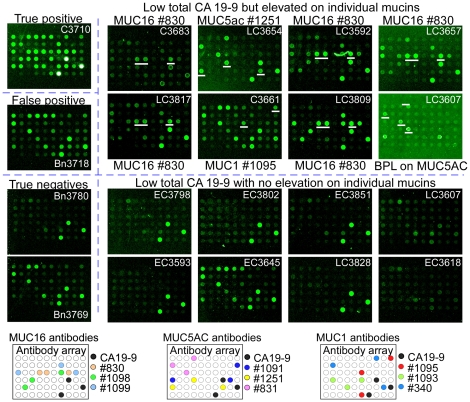
Diversity in CA 19-9 levels on individual proteins. Raw antibody images are shown for patient samples representing diverse marker patterns. Data from sample set 3 (replicate 1) are presented. A cancer sample (labeled ‘True positive’) and pancreatitis sample (labeled ‘False positive’) that were high in total CA 19-9 (above a 75% specificity threshold) are in the top left, and pancreatitis samples that were low in total CA 19-9 (‘True negatives’) are in the bottom left. Cancer samples that were low in total CA 19-9 are grouped by relatively high or low signal at one of the mucins in the top right and bottom right, respectively. The sample identifier is given within each array. In the subgroup picked up by the panel (top-right), the antibody showing elevation in a given sample is listed adjacent to each array. The corresponding antibody spots are underlined in white. Two arrays for sample LC3607 are shown, one detected with BPL (rightmost column, row 2), and the other detected with CA19-9 (rightmost column, row 3). All other arrays were detected with CA19-9. The bottom panels show maps of antibodies targeting MUC16 (left), MUC5AC (middle), and MUC1 (right).

The primary images from selected samples provided insights into the diversity between samples in the carrier proteins that display the CA 19-9 antigen (Fig. 3). The amount of signal at the various capture antibodies gives an indication of the proteins where the CA 19-9 antigen is found. In samples with clearly elevated CA 19-9 (above a 75% specificity threshold), most of the mucin proteins captured here display CA 19-9. Among the samples with total CA 19-9 levels below a 75% specificity threshold, about half show that at least one of the mucins captured here display the CA 19-9 antigen (the prominent mucin carriers are indicated). Other samples show discernable total CA 19-9 but show that these mucins are not carriers of the antigen, and a smaller subset shows no detectable total CA 19-9. Similar subgroups were found in Sets 2 and 3 (not shown), and Western blot analysis confirmed these patterns of CA 19-9 distribution in selected plasma samples (Fig. S2). These findings support the concepts that mucins are major carriers of the CA 19-9 antigen even in low total CA 19-9 states; that diversity exists between people in which mucins carry the antigen; and that other protein besides the mucins probed here carry the CA 19-9 antigen in some patients.

The possibility of detecting other glycans to complement the CA 19-9 antigen was suggested by the primary images (Fig. 3). The samples had been run with detection using the *Bauhinea Purpurea* lectin (BPL) and Wheat Germ Agglutinin (WGA) as a preliminary look at other glycans besides the CA 19-9 antigen. One of the cancer samples that showed negligible signal at any antibody using CA 19-9 detection (sample LC3607) showed clear signal at the MUC5AC antibody using detection with BPL. This result indicates that the MUC5AC mucin is present in the sample and that it does not carry the CA 19-9 antigen, but that it may be detected using another glycan. Although a preliminary result from a single patient, this comparison suggests the importance of detecting other glycans besides the CA19-9 antigen for further performance improvement, especially in the cancer patients with no CA19-9 present.

Because no single protein is the dominant cancer-specific carrier of the CA 19-9 antigen, the detection of CA 19-9 on any of these individual proteins does not out-perform total CA 19-9. However, for individual patients, the detection of the CA 19-9 antigen on the predominant cancer-associated carrier for that patient may give improved discrimination of benign from malignant disease, relative to the total CA 19-9 assay. A panel of such markers, in which each member of the panel detects a subgroup of patients elevated in a certain carrier protein, could thus yield improved performance.

### Improved accuracy using a panel of CA 19-9 detection on individual proteins

**Figure 4:**
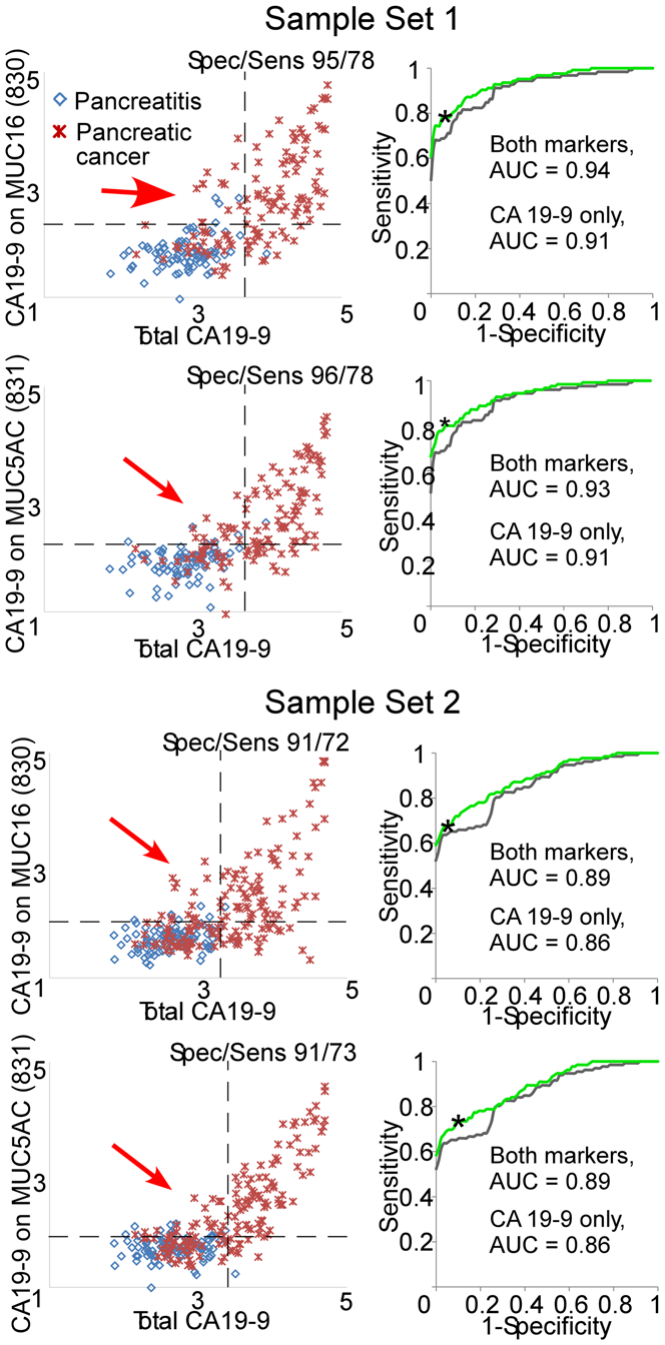
Correlations and complementarity between total CA 19-9 and CA 19-9 on MUC16 and MUC5AC. Each scatter plot compares the values for total CA 19-9 (x axis) to the values for CA 19-9 on MUC16 or MUC5AC. Each point is an individual sample. Samples from Set 1 are presented at top, and samples from Set 2 are presented in the bottom panels. The dashed lines indicate representative thresholds for each marker. The sensitivity and specificity given in each graph represents the performance at those thresholds if a sample exceeding either threshold is called a “case.” The red arrows indicate the samples that are not elevated in total CA 19-9 but are elevated in CA 19-9 on an individual protein. Each ROC curve shows the performance of CA 19-9 alone and the combination of CA 19-9 with the indicated marker. If a sample was elevated in either marker, it was called a “case.” The asterisk indicates the performance at the thresholds in the scatter plots.

The above observations led to the investigation of whether CA 19-9 on individual proteins could complement total CA 19-9 measurements for improved biomarker performance. The relationship between the measurements of total CA 19-9 and CA 19-9 on certain individual proteins showed this possibility (Fig. 4). In some cases, patients that were low in total CA 19-9 were distinguishable from pancreatitis patients by their CA19-9 level on MUC16 or MUC5AC. MUC1 did not show this relationship (not shown). Thresholds could be set by which several cancer patients but no pancreatitis patients were elevated in either CA 19-9 on MUC5AC or CA 19-9 on MUC16 but not in total CA 19-9. Using a combination rule in which an elevation (above the threshold determined individually for each marker) in either total CA 19-9 or CA 19-9 on an individual protein indicated a “case,” and a lack of elevation in both markers indicated a “control,” the combined markers had better performance both sample sets 1 and 2. This improvement was consistent in the repeats of set 1, with the same samples elevated only in one marker or the other (not shown). The improved area-under-the-curve in receiver-operator-characteristic analysis was not statistically significant (p>0.05) in either set. However, the consistent observation of this improvement for two different proteins, in two sample sets from different institutions, and in repeat analyses supports the generality of the finding.

**Figure 5:**
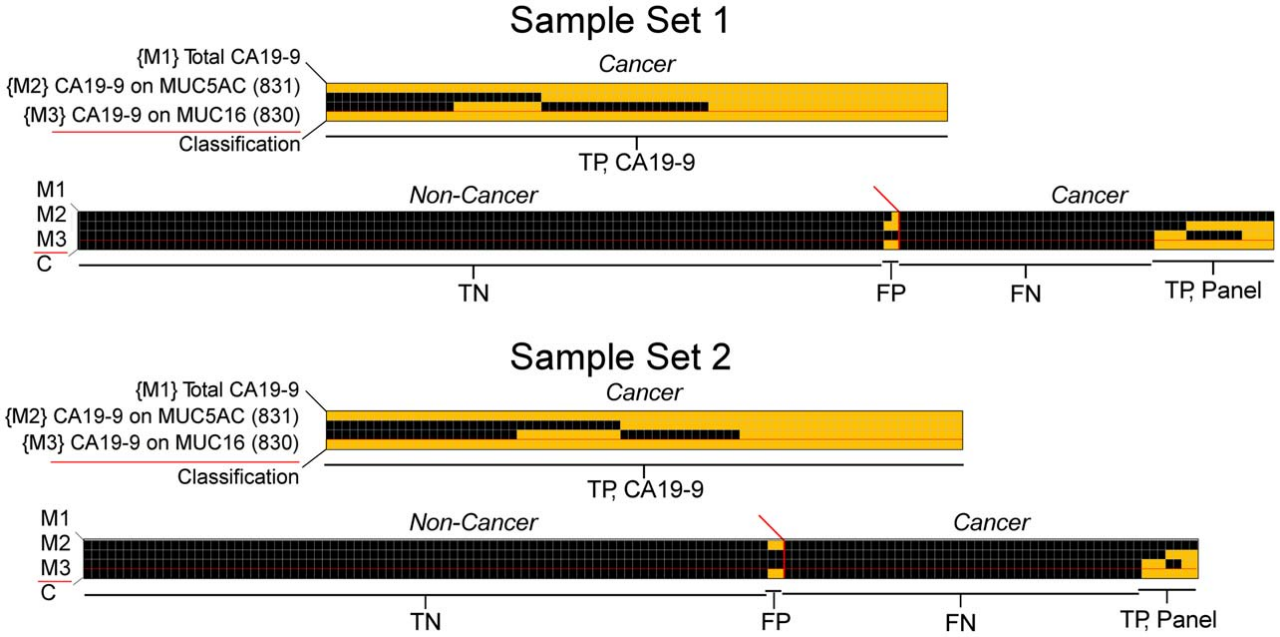
Improved classification over CA 19-9 using a three-marker panel. Each column represents data from a patient sample and each row represents a marker, with the bottom row indicating the patient classification. A threshold was set for each marker, and a yellow square indicates the sample was above the threshold for that marker, and black indicates below the threshold. In the final row, a yellow square indicates the sample was elevated in any of the three markers and classified as a “case.” The true positive (TP) cancer cases that were elevated in CA 19-9 are indicated by ‘TP, CA 19-9’, and the true positive cases elevated only in the other markers are indicated by ‘TP, Panel.’ The false negative (FN) cancer cases are indicated by ‘FN,’ the false positive (FP) control cases that were elevated in a marker are indicated by ‘FP,’ and the true negative (TN) control cases that were low in all markers are indicated by ‘TN.’ Data from Sample Set 1 is presented at top, and data from Sample Set 2 is below.

We next asked whether MUC5AC and MUC16 could be used together with total CA 19-9 to give additional improvement in discriminating cases from controls. The three markers were combined by defining a “case” as having an elevation in at least one of the three markers and a “control” as being low in all three markers. For such a combination rule, the thresholds for each marker need to be individually set to give the best combined performance. We scanned through the possible combinations of thresholds for the three markers that would give a minimum specificity of 98% (two false positives), which was chosen to reveal cancer-specific patterns. A set of thresholds was achieved in which most patients were elevated in total CA 19-9 and another, smaller group was elevated in either CA 19-9-MUC5AC or CA 19-9-MUC16 (Fig. 5). In sample set 1, 11 of the 40 patients that were not elevated in total CA 19-9 were elevated in CA19-9-MUC5AC, and eight were elevated in CA19-9-MUC16. A total of 15 patients were detected by the panel that were not detected by the standard CA 19-9 assay. In sample set 2, seven of the 51 patients low in total CA 19-9 were elevated in either CA19-9-MUC5AC or CA19-9-MUC16. At a specificity of 98%, sensitivity improved from 68% to 80% in sample set 1 and from 61 to 67% in sample set 2 (Table 2). This approach also shows improvements at other specificities; if the thresholds are set to a more permissive 75% specificity, a panel detects seven of 15 patients that were low in total CA 19-9 in sample set 1 (Fig. S3).

**Table 2:** Comparison of the performance of total CA 19-9 and the marker panel in each set.

	Set 1	Specificity	Sensitivity	Accuracy
All samples	CA 19-9 alone	98% (103/105)	63% (79/125)	79%
	Panel	98% (103/105)	74% (93/125)	85%
Early stage	CA 19-9 alone	98% (103/105)	54% (29/54)	83%
	Panel	98% (103/105)	59% (32/54)	85%
Late stage	CA 19-9 alone	98% (103/105)	70% (50/71)	86%
	Panel	98% (103/105)	86% (61/71)	93%

	Set 2			
All samples	CA 19-9 alone	98% (86/88)	56% (74/132)	73%
	Panel	98% (86/88)	67% (88/132)	79%
Early stage	CA 19-9 alone	98% (86/88)	42% (25/60)	75%
	Panel	98% (86/88)	52% (31/60)	79%
Late stage	CA 19-9 alone	98% (86/88)	68% (49/72)	84%
	Panel	98% (86/88)	79% (57/72)	89%

Standard CA 19-9 had a lower sensitivity for early-stage cancer than late-stage cancer (Table 2), so we examined whether the improvement using the panel held true for both early and late stages. A direct comparison was achieved by adjusting the threshold of standard CA 19-9 to give the same specificity (98%) as the panel. Of the 14 patients detected by the panel in set 1 that were not detected by CA 19-9 alone, just three were early stage, and of the 14 additional patients detected by the panel in Set 2, six were early stage (Table 2). Therefore, the panel has the potential of improving the detection of early-stage cancer relative to the standard CA 19-9 assay, but it is similar to standard CA 19-9 in that it detects a higher percentage of late-stage cancer than early-stage cancer.

While it was a relatively small subset of CA 19-9-low patients that were picked up by the panel, the marker patterns were consistent between the independent sample sets. In both sets 1 and 2, about a third of the patients detected by the panel were elevated in only CA19-9-MUC5AC, another third elevated in only CA19-9-MUC16, and another third elevated in both. The consistency between the sample sets in the overall results supports that the various patterns of marker expression, high in all or high in individual members of the panel, represent biological subgroups that may be observed in the larger population.

The use of three independent sets also gave information about the relative merits of serum and plasma, since sample set 1 was plasma and sets 2 and 3 were serum. The same markers were found to be effective between sets 1, 2, and 3, with similar relationships to total CA 19-9. That result suggests that the relative levels between cases and controls are not greatly affected by the mode of preparation of the sample. In addition, the reproducibility of the measurements was similar between serum and plasma. Each data set included repeated series of dilutions of pooled samples. At a 10-fold dilution of the pool, representing concentrations similar to the individual sample, the coefficient of variation between the replicate measurements was 23% for the serum samples (from Set 2) and also 23% for the plasma samples (from Set 3) (data not shown). That finding suggests that the stability of the markers is not greatly affected by whether the samples are prepared as serum or plasma.

## Discussion

The need for improved blood markers for pancreatic cancer is great. Such markers would have important applications in the detection and diagnosis of the disease, leading to improved patient management and outcomes. The sub-optimal performance of the CA 19-9 assay may, in some cases, be due to the appearance of the CA 19-9 carbohydrate antigen on carrier proteins that are not specific to cancer. By detecting the antigen specifically on the proteins that are the predominant carriers in cancer, improved performance may result. We examined this possibility using antibody arrays with glycan detection, which provided a convenient approach to measuring the CA 19-9 antigen on multiple, individual proteins. We found that the mucins MUC1, MUC5AC, and MUC16 are indeed major cancer-associated carriers of CA 19-9, but because of the diversity among patients in the proteins that carry CA 19-9, the detection of CA 19-9 on any single protein did not out-perform total CA 19-9. However, for individual patients with low CA 19-9 in which a predominant carrier was identified, selective discrimination from the pancreatitis controls was possible. A combination marker comprising total CA 19-9 plus CA 19-9 on selected proteins could yield improved sensitivity of cancer detection over total CA 19-9 alone. Similar results were observed in two independent sample sets from two different institutions. This work demonstrates the potential of improving detection accuracy using glycan measurements on individual proteins.

A new biomarker to more sensitively detect cancer relative to benign disease conditions could be significant in a variety of ways. A possible area of application would be to diagnose patients that have pancreatic abnormalities as discovered by CT scan. Several conditions in addition to malignancies produce abnormal pancreatic findings by CT [16], such as cystic lesions, pancreatitis, and common bile duct obstruction, and only some require further intervention. Because no molecular marker exists to sort out the conditions, nearly all patients go on to endoscopic ultrasound and potential biopsy. A reduction in this invasive, costly, and risky procedure is desirable, considering the high rate of patients with benign conditions that receive it. The patients that might benefit most from markers based on this strategy would be those with CA 19-9 levels that are below a threshold for disease-specific elevation but above the analytical detection limit of the assay. For those patients, it may be possible to accurately determine who should be referred for additional diagnostic workup.

Future work in the development of a biomarker includes further validating and characterizing the improved sensitivity of the current marker panel and determining the panel's ability to meet the performance needs of specific clinical applications. The most effective validation will make use of samples that were collected in the clinical setting and patient population intended for eventual use, in this case patients with pancreatic abnormalities who are being considered for referral for further diagnostic workup. In addition, it will be important to develop clinical assays for these markers. Clinical assays would ensure lack of interference from potentially confounding factors and would provide the precision and control over variability that are required to fully assess marker performance.

Further biomarker discovery could be targeted to the subgroup not detected by the panel (Fig. 5). For patients that may have weak levels of the CA 19-9 antigen, yet the main protein carrier of the antigen is unknown, it would be valuable to identify the predominant carrier of the antigen. New assays CA 19-9 on that protein could provide selective detection of those patients, as demonstrated here. For patients that have undetectable CA 19-9 levels, the detection of another glycan on the mucins or some other protein carrier may provide discrimination. This situation was present in patient 3607 (Fig. 3). This patient showed no CA 19-9 signal on any carrier but showed strong signal at the MUC5AC capture antibody when detected by the lectin BPL. The glycan bound by BPL, terminal beta-linked galactose, is distinct from the glycan bound by CA 19-9, confirming the need for the detection of additional glycans beyond CA 19-9. This result is consistent with the fact that certain individuals, estimated to be around 5% of the population, are genetically deficient in an enzyme that completes a critical step in the biosynthesis of the CA 19-9 antigen [17], [18]. Finally, for patients without detectable CA 19-9 or mucin, proteins and glycans must be sought.

Improving the limit of detection of the analytical assay may also enhance the ability to detect the cancer patients. Some of the patients not detected in this study may have mucin proteins secreted into the blood but at very low levels, which might be detectable given a very sensitive assay. This point may be especially important for early-stage cancer patients, which are likely to have lower concentrations of tumor markers. Our data show that we detect a subset of early-stage cancer patients (Table 2 and Fig. 5) but that late-stage patients are more frequently elevated. Several options are available for improving the detection limits of the assay. Amplification of the fluorescence signal is possible using rolling-circle amplification [19], [20] or tyramide signal amplification [21]. A novel format that restricts the sample to ultra-low detection volumes can lower detection limits using enzyme-based chemiluminescence detection [22]. A new generation of electrochemical biosensors is achieving or surpassing detection limits achieved by fluorescence [23], which provides another possible route for the improved detection of cancer patients.

The subgroups identified in this work may represent biologically distinct subgroups of pancreatic cancer that have clinical implications. Studies of cancers of other organs have identified subcategories of disease defined by molecular characteristics [24], but clear subcategories of pancreatic cancer have not emerged despite the gene expression and molecular profiling studies that have been performed on pancreatic ductal adenocarcinoma [25], [26], However, it is likely that defined subgroups of the disease exist that have distinct molecular characteristics and that produce distinct alterations in the blood. Future work will investigate connections between distinct blood marker profiles and other information about the tumor or patient. Given the roles of the Lewis family of carbohydrate structures (of which the CA 19-9 antigen is a member) in modulating immunological and vascular interactions [27], [28], the possibility exists that differences in the carrier protein of the CA 19-9 antigen would contribute to distinct courses of tumor progression. For example, because sialyl LewisA is a ligand for selectin receptors that initiate lymphocyte interactions with vascular walls, high levels in the blood may modulate inflammatory responses [27]. The modulation of mucin function through altered glycosylation also might have biological implications. The mucins have normal functions in the protection and control of epithelial surfaces [11], [29], and the increased presence of Lewis antigens on mucins could have significant physiological effects both in the local tumor environment and at distant sites accessed through the lymph and circulation. Because the CA 19-9 antigen is sialylated, mucins bearing that glycan would not be cleared through the asialoglycoprotein receptors on liver cells, allowing mucin levels to increase and remain high in the circulation of cancer patients. Some evidence of direct immunomodulatory effects of tumor-derived mucins on leukocytes has been uncovered [30], [31], [32].

The approach to biomarker development demonstrated here may be useful in other biomarker applications. The detection of glycans on specific proteins may yield greater accuracy for a variety of disease states than by detecting just protein levels, as with standard immunoassays, or just the levels of a particular glycan on all proteins, as with the conventional CA 19-9 assay. The antibody-lectin sandwich array provides an ideal format for testing combinations of proteins and glycans for such investigations [33]. The proteins and glycans to be targeted on the arrays can be derived from known molecular alterations, such as mucins in pancreatic cancer [11], [29], [34], or from genomics, proteomics, and glycoproteomics studies. Glycoproteomics methods used in combination with antibody arrays could represent a powerful strategy for biomarker development [35], the former providing potential new proteins and glycans to test, and the latter providing an efficient and accurate means of testing multiple candidates. Potential additional areas of application include screening for colon cancer, which displays mucin and glycan alterations, and the early detection of incipient cancer in chronic inflammatory situations. It will be valuable to map the tissue-specificity of protein carriers of cancer-associated glycans, which will increase the information content of the assay.

In summary, an improvement over the conventional CA 19-9 assay may be achievable by detecting the CA 19-9 antigen on specific proteins rather than on all protein carriers. The identification of subgroups of patients based on CA 19-9 carrier status suggests biologically distinct entities of the disease that will be will be optimally detected by complementary markers. Using a combination of total CA 19-9 and CA 19-9 on individual proteins, the sensitivity of cancer detection was improved relative to CA 19-9 alone in two independent sample sets from two different institutions, achieving 67-80% sensitivity at 98% specificity. The expansion of this panel with additional glycans and protein carriers should further improve performance. Validation will be performed using blinded samples collected from the setting of the intended clinical application, in accordance with the developed standards for biomarker validation [36].

## Materials and Methods

### Ethics statement

All sample collection and research was conducted under protocols approved by the Institutional Review Boards at Evanston Northwestern Healthcare, the University of Michigan Medical School, the University of Pittsburgh School of Medicine, and the Van Andel Research Institute. Written, informed consent was obtained from all participants in the study.

### Serum and plasma samples

Serum samples from Evanston Northwestern Healthcare and the University of Michigan Medical School and plasma samples (using EDTA as the anti-coagulant) from the University of Pittsburgh School of Medicine were collected from pancreatic cancer, pancreatitis and healthy subjects (Table 1). Early-stage cancer was defined as stages I and II, and late-stage cancer was defined as stages III and IV. The pancreatitis patients were a mixture of chronic and acute. The control subjects were healthy with no evidence of pancreatic, biliary or liver disease. The samples at each site were collected using a standard operating procedure based on the serum and plasma protocols from the Early Detection Research Network. All samples were stored at −80°C and sent frozen on dry ice. Each aliquot had been thawed no more than three times before use.

### Antibodies and lectins

The antibodies and lectins were obtained from various sources (see Tables S1, S2, S3). All antibodies were screened for reactivity and integrity using Western blots, purified, and prepared at 0.5 mg/ml in pH 7.2 phosphate-buffered saline (PBS) for non-contact array printer and at 0.25 mg/ml in pH 7.2 PBS for contact array printer. The steps of antibody purification included ultracentrifugation at 47,000 g at 4 degrees for 1 hour and dialysis (Slide-A-Lyzer Mini Dialysis Units, Pierce Biotechnology) against pH 7.2 PBS at 4 degree for 2 hours.

### Microarray fabrication

Approximately 170 pg (350 pl at 500 µg/ml or 700 pl at 250 µg/ml) of each antibody was spotted on the surfaces of ultra-thin nitrocellulose-coated microscope slides (PATH slides, GenTel Biosciences) by a non-contact microarrayer (sciFLEXARRAYER, Scienion) performed at GenTel Biosciences (Madison, WI) for the slides used in replicates 1 and 2 of sample set 1, and by a contact microarrayer (2470, Aushon Biosciences) for the rest of the experiments. Forty-eight identical arrays containing triplicates of all antibodies were printed on each slide. Hydrophobic borders were imprinted around each array using a stamping device (SlideImprinter, The Gel Company, San Francisco, CA).

### Microarray assays

Microarray sandwich assays were performed to measure either the level of total CA19-9 or the glycan levels on the proteins captured by the immobilized antibodies (Fig. 1a). The sandwich assay consisted of four 1-hour-incubations in room temperature (RT) with the following reagents: 1) blocking buffer (PBS containing 0.5% Tween-20 (PBST0.5) and 1% BSA); 2) a serum or plasma sample, diluted two-fold in 1×TBS containing 0.08% Brij, 0.08 Tween-20, 50 µg/ml protease inhibitor cocktail (Complete Protease Inhibitor Tablet, Roche Applied Science), and a cocktail of IgG from mouse, goat, and sheep each at 100 µg/ml and rabbit IgG at 200 µg/ml (Jackson ImmunoResearch Laboratories, Inc.); 3) biotinylated detection antibody or lectin (2 µg/ml), diluted in PBST0.1 containing 0.1% BSA; 4) streptavidin-phycoerythrin (10 µg/ml, Roche Applied Science), diluted in PBST0.1 containing 0.1% BSA. After each step, the slides were rinsed in three baths of PBST0.1 and dried by centrifugation (Eppendorf 5810R, rotor A-4-62, 1500× g).

The measurement of glycans by using lectins detection on the captured proteins (Fig. 1a) was carried out as above, except the glycans on the spotted antibodies were derivatized to prevent lectin binding to the antibodies [12], and the arrays were probed with glycan-binding lectins. Fluorescence emission from the phycoerythrin was detected at 570 nm using a microarray scanner (LS Reloaded, Tecan). All arrays within one slide were scanned at a single laser power and detector gain setting. The images were quantified using the software program GenePix Pro 5.0 (Molecular Devices, Sunnyvale, CA). Spots were identified using automated spot-finding or manual adjustments for occasional irregularities. The median local backgrounds were subtracted from the median intensity of each spot, and triplicate spots were averaged using the geometric mean. The coefficient of variation between replicate analyzed spots was typically under 10%.

### Statistical analyses and software

Pearson correlations, Student's T-tests, and receiver-operator characteristic analyses were calculated using Microsoft Excel. The scatter and box plots were created using OriginPro 8, and figure production was performed using Canvas X.

## Supporting Information

Figure S1 **Selection of antibodies that capture CA 19-9 carrier proteins.** a) 12 serum samples (6 benign+6 cancer) were incubated on arrays containing 58 different capture antibodies at detected with the CA 19-9 antibody. A series of steps were taken to select antibodies that capture CA 19-9 carrier proteins and that have potential value in subsequent experiments. i) Antibodies were removed that produced consistently low signal across all samples, defined as an average fluorescence across all samples of <1.5 time the fluorescence in the PBS negative control array. ii) Next, we removed antibodies that produced signals with a very low standard deviation across samples, since a lack of change between samples would not produce valuable information in later experiments. The threshold was <400 RFU. iii) Finally, we compared signals between the pancreatic cancer sera and the control pancreatitis sera to identify antibodies potentially showing differences between the groups using the Mann-Whitney test. Since this was a preliminary analysis the significance threshold was set at α = 0.10 b) The process was repeated for 16 of the most promising antibodies from the first run, using a set of 44 serum samples (4 healthy+20 benign+20 cancer). The more powerful student's t-test was used due to the larger sample size, but with a more stringent α = 0.05. c) Fluorescence values across the case and control samples for two of the best capture antibodies, anti-MUC5AC and anti-MUC16.
(TIF)

Figure S2 **CA 19-9 immunoblots of selected samples.** Of fundamental interest is the distribution of CA 19-9 carrier proteins in these subgroups. An approach to visualize the range of proteins carrying the CA 19-9 antigen is to fractionate the plasma proteins using SDS-PAGE and immunoblot for the CA 19-9 antigen, which we did for representative samples from the subgroups defined by CA 19-9 carrier protein status. The indicated plasma samples from Set #1 were fractionated on a 4–12% gradient polyacrylamide gel and probed by Western blot using the CA 19-9 antibody. The samples that were high in CA 19-9 by microarray showed a broad range of molecular weights with high signal, indicating many proteins containing the CA 19-9 antigen. The samples that were below the 75% specificity threshold but that showed significant signal at the mucin proteins showed only faint bands at high molecular weights (>150 kD); and the samples not detected by any marker showed no discernable or only faint bands. This results shows that no major protein carriers of the CA 19-9 antigen, at least in the molecular weights observed in this format, are present in the low CA 19-9 samples. Thus, the identification of cancer in the remaining samples not picked up by the panel most likely will rely on additional proteins or glycans.
(TIF)

Figure S3 **Increased sensitivity using markers complementary to total CA 19-9.** Data from sample set 1, replicate 1 are presented. a) Comparison of CA19-9 on MUC16 to total CA19-9. The levels of CA 19-9 on MUC16 for each sample are plotted along the vertical axis, and the total CA 19-9 levels for the same samples are plotted along the horizontal axis. The plot shows only the lower 50% of the samples by total CA 19-9. The vertical line indicates the threshold defined to give 75% specificity by total CA19-9. The horizontal dashed line indicates a threshold for CA19-9 on MUC16 which would result in the detection of additional cancer samples (noted by the arrows) without detecting additional pancreatitis samples. b) Combined results of total CA19-9 and four additional complementary markers. The samples are ordered in the columns (Bn is benign, EarlyC is early-stage cancer, LateC is late-stage cancer, Cancer is unknown stage cancer) and the markers in the rows. The threshold for total CA19-9 was set to 75% specificity, and the threshold for each additional marker was defined as in panel a. A yellow square indicates a measurement above the threshold, a black square indicates below the threshold, and gray squares are missing data. The blue box denotes the cancer samples not detected by CA 19-9 (CA 19-9 measurements in the red box). The samples picked up by the additional markers are highlighted by blue column labels.
(TIF)

Table S1 **Antibodies used in the large-scale screening for CA 19-9 carrier proteins.**
(DOCX)

Table S2 **Antibodies used for the follow up experiments in screening for CA 19-9 carrier proteins.**
(DOCX)

Table S3 **Antibodies used on the arrays and for detection in the biomarker profiling experiments.**
(DOCX)
