# Supplementary figures and images for: Enhanced Discrimination of Malignant from Benign Pancreatic Disease by Measuring the CA 19-9 Antigen on Specific Protein Carriers
Source: PLoS One. 2011 Dec 29;6(12):e29180. doi: 10.1371/journal.pone.0029180 (PMC3248411; doi:10.1371/journal.pone.0029180)

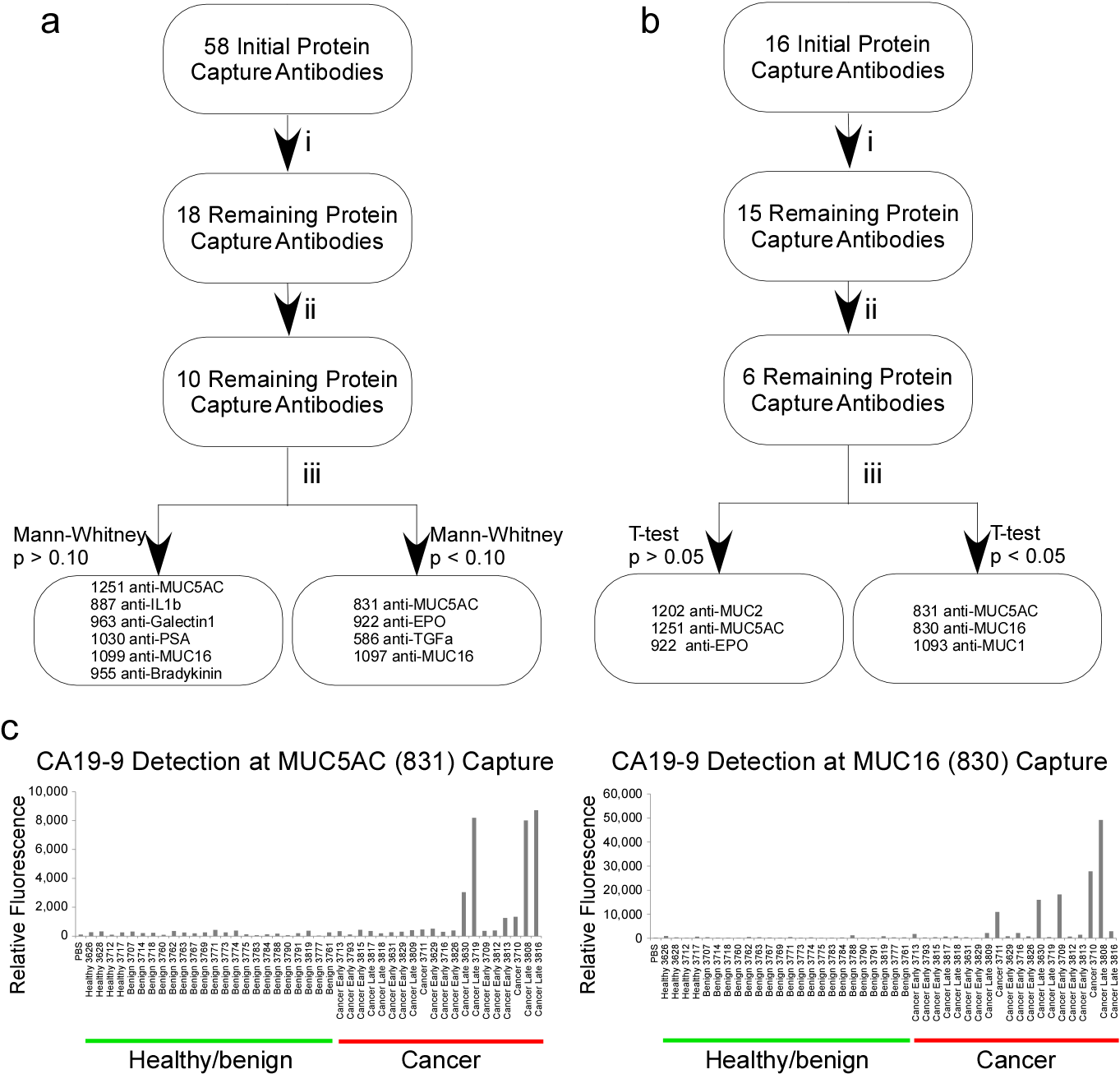

Supplement: Figure S1 — Selection of antibodies that capture CA 19-9 carrier proteins. a) 12 serum samples (6 benign+6 cancer) were incubated on arrays containing 58 different capture antibodies at detected with the CA 19-9 antibody. A series of steps were taken to select antibodies that capture CA 19-9 carrier proteins and that have potential value in subsequent experiments. i) Antibodies were removed that produced consistently low signal across all samples, defined as an average fluorescence across all samples of <1.5 time the fluorescence in the PBS negative control array. ii) Next, we removed antibodies that produced signals with a very low standard deviation across samples, since a lack of change between samples would not produce valuable information in later experiments. The threshold was <400 RFU. iii) Finally, we compared signals between the pancreatic cancer sera and the control pancreatitis sera to identify antibodies potentially showing differences between the groups using the Mann-Whitney test. Since this was a preliminary analysis the significance threshold was set at α = 0.10 b) The process was repeated for 16 of the most promising antibodies from the first run, using a set of 44 serum samples (4 healthy+20 benign+20 cancer). The more powerful student's t-test was used due to the larger sample size, but with a more stringent α = 0.05. c) Fluorescence values across the case and control samples for two of the best capture antibodies, anti-MUC5AC and anti-MUC16. (TIF) [file pone.0029180.s001.tif]

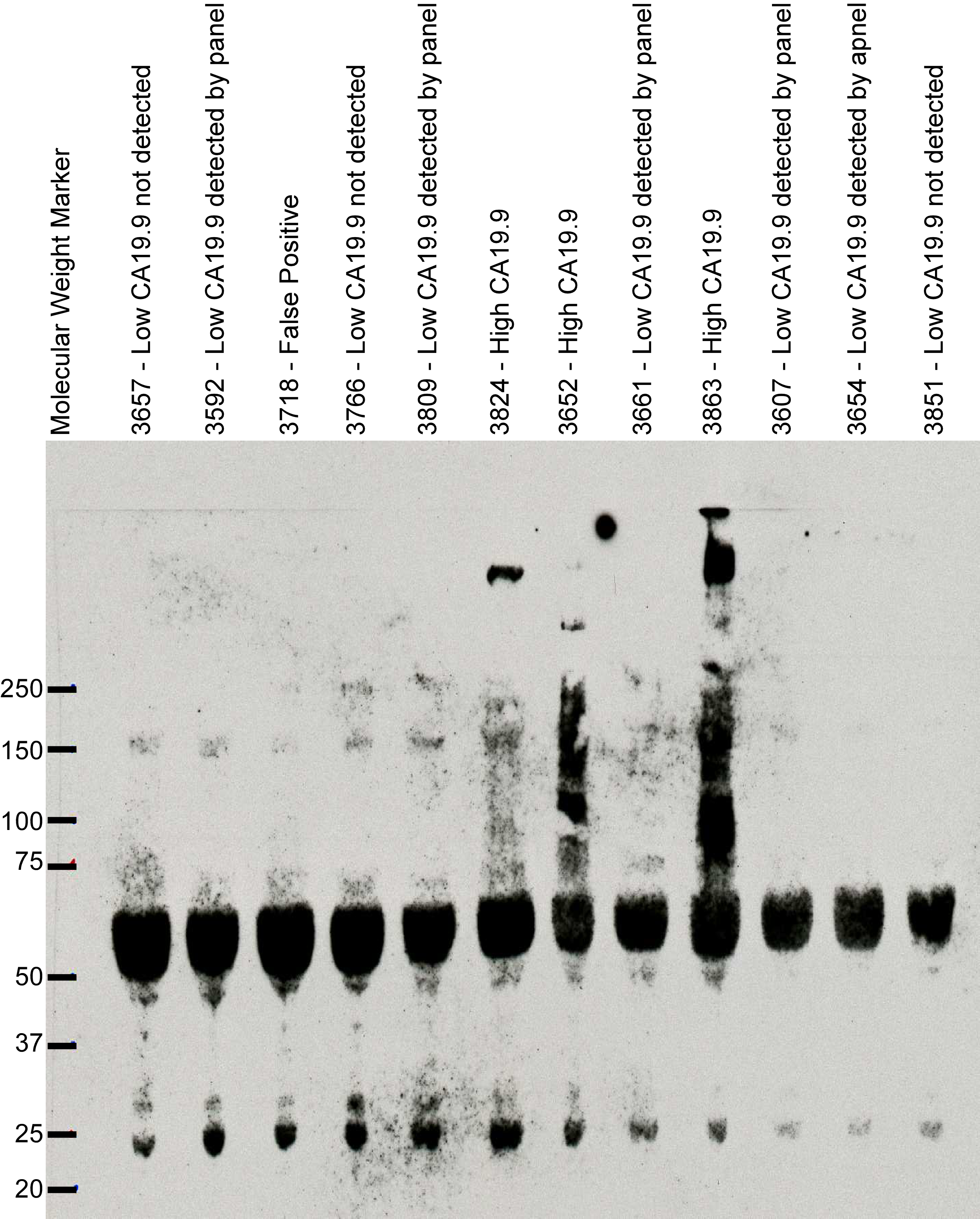

Supplement: Figure S2 — CA 19-9 immunoblots of selected samples. Of fundamental interest is the distribution of CA 19-9 carrier proteins in these subgroups. An approach to visualize the range of proteins carrying the CA 19-9 antigen is to fractionate the plasma proteins using SDS-PAGE and immunoblot for the CA 19-9 antigen, which we did for representative samples from the subgroups defined by CA 19-9 carrier protein status. The indicated plasma samples from Set #1 were fractionated on a 4–12% gradient polyacrylamide gel and probed by Western blot using the CA 19-9 antibody. The samples that were high in CA 19-9 by microarray showed a broad range of molecular weights with high signal, indicating many proteins containing the CA 19-9 antigen. The samples that were below the 75% specificity threshold but that showed significant signal at the mucin proteins showed only faint bands at high molecular weights (>150 kD); and the samples not detected by any marker showed no discernable or only faint bands. This results shows that no major protein carriers of the CA 19-9 antigen, at least in the molecular weights observed in this format, are present in the low CA 19-9 samples. Thus, the identification of cancer in the remaining samples not picked up by the panel most likely will rely on additional proteins or glycans. (TIF) [file pone.0029180.s002.tif]

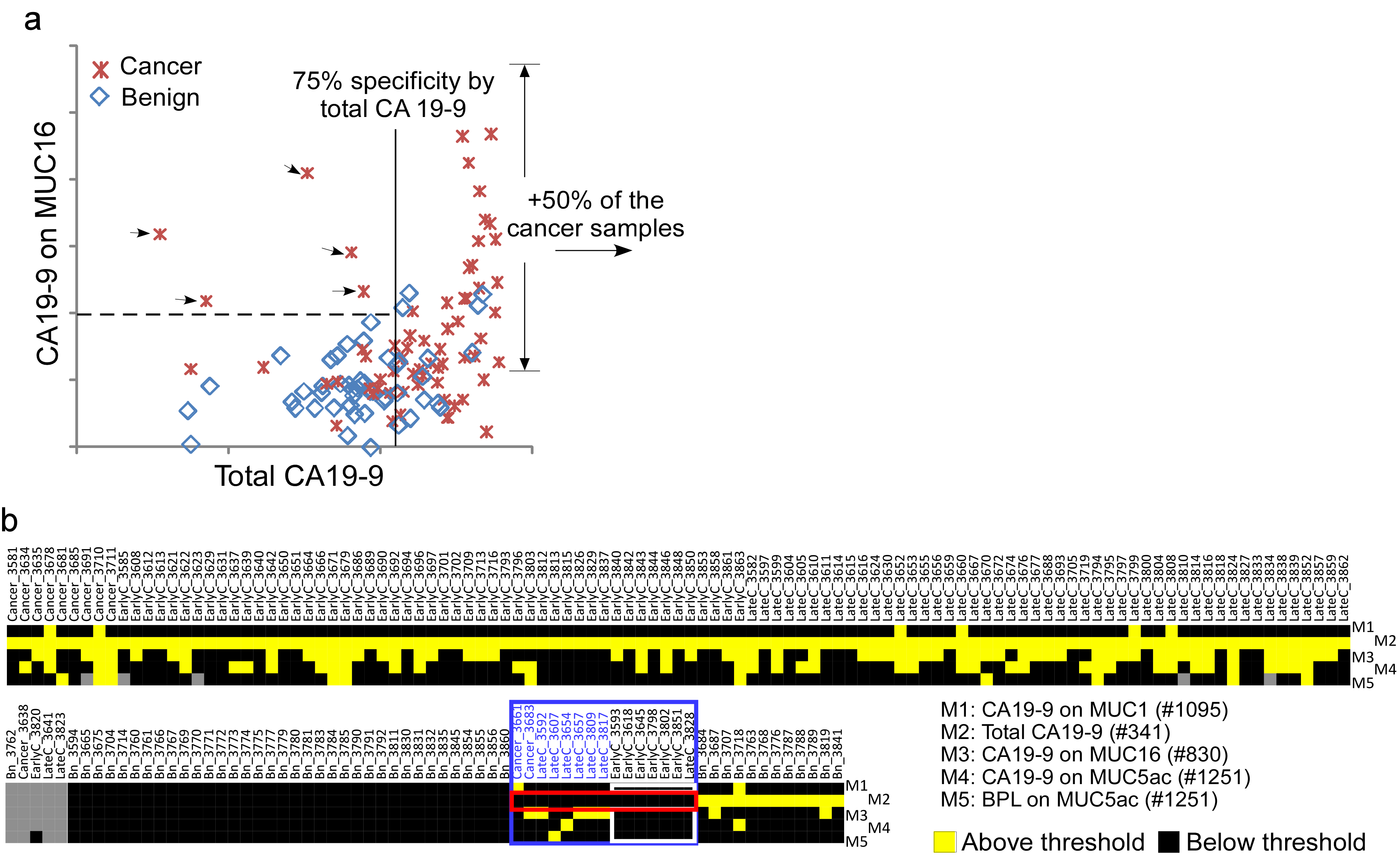

Supplement: Figure S3 — Increased sensitivity using markers complementary to total CA 19-9. Data from sample set 1, replicate 1 are presented. a) Comparison of CA19-9 on MUC16 to total CA19-9. The levels of CA 19-9 on MUC16 for each sample are plotted along the vertical axis, and the total CA 19-9 levels for the same samples are plotted along the horizontal axis. The plot shows only the lower 50% of the samples by total CA 19-9. The vertical line indicates the threshold defined to give 75% specificity by total CA19-9. The horizontal dashed line indicates a threshold for CA19-9 on MUC16 which would result in the detection of additional cancer samples (noted by the arrows) without detecting additional pancreatitis samples. b) Combined results of total CA19-9 and four additional complementary markers. The samples are ordered in the columns (Bn is benign, EarlyC is early-stage cancer, LateC is late-stage cancer, Cancer is unknown stage cancer) and the markers in the rows. The threshold for total CA19-9 was set to 75% specificity, and the threshold for each additional marker was defined as in panel a. A yellow square indicates a measurement above the threshold, a black square indicates below the threshold, and gray squares are missing data. The blue box denotes the cancer samples not detected by CA 19-9 (CA 19-9 measurements in the red box). The samples picked up by the additional markers are highlighted by blue column labels. (TIF) [file pone.0029180.s003.tif]
