# Supplementary material for: Enhanced Discrimination of Malignant from Benign Pancreatic Disease by Measuring the CA 19-9 Antigen on Specific Protein Carriers
Source: PLoS One. 2011 Dec 29;6(12):e29180. doi: 10.1371/journal.pone.0029180 (PMC3248411; doi:10.1371/journal.pone.0029180)
Supplement: Table S1 — Antibodies used in the large-scale screening for CA 19-9 carrier proteins. (DOCX) [file pone.0029180.s004.docx]

Table S1. Antibodies used in the large-scale screening for CA 19-9 carrier proteins

| **ID** | **Antibody** | **Species** | **Type** | **Source** | **Clone #** | **Catalog #** |
| --- | --- | --- | --- | --- | --- | --- |
| 194 | Anti Von Willebrand Factor | *Orytolagus cuniculus* | Ig | DAKO | NA | A0082 |
| 254 | Anti Tumor Necrosis Factor | *Mus musculus* | IgG | Lucien Aarden, U. Amsterdam | CLB-TNF/7 | NA |
| 269 | Anti Interleukin 6 | *Mus musculus* | IgG | Sigma-Aldrich | 6708.11 | I7901 |
| 275 | Anti beta lipoprotein | *Orytolagus cuniculus* | Ig | Biotrend | NA | 5685-3010 |
| 298 | Anti Insulin-like Growth Factor Binding Protein 3 | *Mus musculus* | IgG | R&D Systems | 84728.111 | MAB305 |
| 340 | Anti Mucin 1 | *Mus musculus* | IgG | USBiological | 1.B.831 | C0050-23 |
| 348 | Anti b2 Microglobulin | *Mus musculus* | IgG | USBiological | 0.N.17 | M3890-05X |
| 351 | Anti Serum Amyloid A | *Mus musculus* | IgG | Abcam | 115 | ab687 |
| 370 | Anti Insulin-like Growth Factor 1 | *Capra hircus* | IgG | R&D Systems | NA | AF-291-NA |
| 432 | Anti Glypican 3 | *Orytolagus cuniculus* | IgG | Santa Cruz Biotechnology, Inc. | NA | sc-11395 |
| 506 | Anti gamma-carboxyglutamyl residues | *Mus musculus* | IgG | American Diagnostica, Inc. | NA | 3570 |
| 526 | Anti Glucagon | *Orytolagus cuniculus* | Ig | Lab Vision Corporation | NA | RB-1422-A1 |
| 527 | Anti Insulin | *Mus musculus* | IgG | Lab Vision Corporation | I-10 | MS-1595-PABX |
| 571 | Anti Matrix Gla Protein | *Mus musculus* | IgG | Alexis Biochemicals | 52.1C5D | ALX-804-512-C100 |
| 580 | Anti Epidermal Growth Factor | *Mus musculus* | IgG | Abcam | EGF-10 | ab10409 |
| 586 | Anti Transforming Growth Factor alpha | *Mus musculus* | IgG | Lab Vision Corporation | MF9 | MS-670-PABX |
| 587 | Anti Angiostatin | *Mus musculus* | IgG | R&D Systems | 79735 | MAB926 |
| 589 | Anti Angiogenin | *Mus musculus* | IgG | Sigma-Aldrich | 14017.7 | A9850 |
| 594 | Anti Platelet Derived Growth Factor B | *Mus musculus* | IgG | R&D Systems | 108132 | MAB220 |
| 653 | Anti Vascular Endothelial Growth Factor | *Mus musculus* | IgG | R&D Systems | 26503 | MAB293 |
| 656 | Anti Interleukin 8 | *Mus musculus* | IgG | R&D Systems | 6217 | MAB208 |
| 660 | Anti Endostatin | *Mus musculus* | Ig | R&D Systems | Unknown | Kit: DY1098 Part: 841455 |
| 675 | Anti Vitronectin | *Mus musculus* | IgG | Biodesign International | BDI215 | N77810M |
| 684 | Anti Mucin 1 | *Mus musculus* | IgG | Abcam | SM3 | ab22711 |
| 686 | Anti Fibronectin | *Capra hircus* | IgG | R&D Systems | NA | AF1918 |
| 698 | Anti Heparin Cofactor 2 | *Capra hircus* | IgG | Haematologic Technologies | NA | PAHCII-G |
| 702 | Anti Interleukin 10 | *Orytolagus cuniculus* | Ig | PeproTech | NA | 900-K21 |
| 716 | Anti Apolipoprotein A-I | *Orytolagus cuniculus* | IgG | CALBIOCHEM | NA | 178422 |
| 769 | Anti Laminin | *Mus musculus* | IgM | USBiological | 5K138 | L1225-01A |
| 793 | Anti Endorepellin | *Capra hircus* | IgG | R&D Systems | NA | AF2364 |
| 821 | Anti Perlecan | *Mus musculus* | IgG | USBiological | 3G166 | H1890-93 |
| 831 | Anti Mucin 5AC | *Mus musculus* | IgG | AbD Serotec | 45M1 | 1695-0128 |
| 887 | Anti Interleukin 1 beta | *Mus musculus* | IgG | R&D Systems | 8516 | MAB201 |
| 909 | Anti alpha 1 Antitrypsin | *Capra hircus* | IgG | Abcam | NA | ab7633 |
| 915 | Anti Transforming Growth Factor beta 1 | *Mus musculus* | IgG | R&D Systems | 9016 | MAB240 |
| 922 | Anti Erythropoietin (Epo-16) | *Mus musculus* | IgG | Stemcell Technologies | 16F1H11 | 1300 |
| 955 | Anti Bradykinin | *Orytolagus cuniculus* | Ig | AbD Serotec | NA | 0100-0443 |
| 963 | Anti mouse Galectin 1 (50% human cross-reactivity) | *Rattus norvegicus* | IgG | R&D Systems | 201002 | MAB12451 |
| 966 | Anti mouse Galectin 3 (50% human cross-reactivity) | *Capra hircus* | IgG | R&D Systems | NA | AF1197 |
| 1030 | Anti Prostate Specific Antigen | *Orytolagus cuniculus* | Ig | Fitzgerald | NA | 20-PR50 |
| 1053 | Anti Mucin 2 | *Mus musculus* | IgG | Abcam | 994/152 | ab22712 |
| 1097 | Anti Mucin 16 | *Mus musculus* | IgG | GeneTex | X75 | GTX10029 |
| 1099 | Anti Mucin 16 | *Mus musculus* | IgG | USBiological | 1.B.826 | C0050-05 |
| 1178 | Anti C-Reactive Protein | *Mus musculus* | IgG | GenWay Biotechnologies | BGN/03/703 | 20-783-73078 |
| 1192 | Anti Mucin 3 | *Mus musculus* | IgG | Abcam | M3.1 | ab24068 |
| 1193 | Anti Mucin 17 | *Orytolagus cuniculus* | IgG | Dr. Surinder Batra, Univ. of Nebraska | SN1139-2 | NA |
| 1204 | Anti Mucin 5B | *Mus musculus* | Ig | Novus Biologicals | NA | H00004587-A01 |
| 1205 | Anti Mucin 3A | *Mus musculus* | IgG | Lifespan Biosciences | Not Listed | LS-C16658 |
| 1223 | Anti SAP | *Mus musculus* | IgG | Abcam | SAP-5 | ab13334 |
| 1224 | Anti KNG | *Mus musculus* | IgG | Dr. Robert Colman, Temple University | 2B5 | NA |
| 1241 | Anti Vimentin | *Capra hircus* | IgG | R&D Systems | NA | AF2105 |
| 1249 | Anti Human Milk Fat Globule 1 | *Mus musculus* | IgG | Thermo Scientific | EDM45 | MS-512-P1 |
| 1251 | Anti Mucin 5AC | *Mus musculus* | IgG | Thermo Scientific | 2-11M1 | MA1-35704 |
| 1271 | Anti Apolipoprotein E | *Capra hircus* | IgG | GeneTex | NA | GTX27620 |
| 1276 | Anti Mucin-like Protocadherin | *Orytolagus cuniculus* | Ig | Sigma Life Science | NA | HPA009173 |
| 1279 | Anti *Bacillus anthracis* Protective Antigen (Negative Control) | *Mus musculus* | IgG | Dr. Brian Cao, VARI | 10F5 | NA |
| 1295 | Anti Cancer Antigen 19.9 | *Mus musculus* | IgG | USBiological | 9L426 | C0075-03A |
| 1297 | Anti Laminin 5 | *Mus musculus* | IgG | Abcam | P3H9-2 | ab78286 |
| 1300 | Anti Versican | *Mus musculus* | IgG | Abcam | MM0600-7D41 | ab89934 |
