# Supplementary material for: Enhanced Discrimination of Malignant from Benign Pancreatic Disease by Measuring the CA 19-9 Antigen on Specific Protein Carriers
Source: PLoS One. 2011 Dec 29;6(12):e29180. doi: 10.1371/journal.pone.0029180 (PMC3248411; doi:10.1371/journal.pone.0029180)
Supplement: Table S2 — Antibodies used for the follow up experiments in screening for CA 19-9 carrier proteins. (DOCX) [file pone.0029180.s005.docx]

Table S2. Antibodies used for the follow up experiments in screening for CA 19-9 carrier proteins

| **ID** | **Antibody** | **Species** | **Type** | **Obtained** | **Clone #** | **Catalog#** |
| --- | --- | --- | --- | --- | --- | --- |
| 1322 | Gala1,3Galb1,4GlcNAc-Sp-BSA | NA | Glycoconjugate | Vector Labs | NA | NGP0334 |
| 1321 | N-acetyllactosamine-BSA | NA | Glycoconjugate | Vector Labs | NA | NGP1201 |
| 1320 | 3'-Sialyl-N-acetyllactosamine-BSA | NA | Glycoconjugate | Vector Labs | NA | NGP1301 |
| 1319 | Lacto-N-fucopentaose III-BSA | NA | Glycoconjugate | Vector Labs | NA | NGP0502 |
| 1318 | Lewis x-BSA | NA | Glycoconjugate | Vector Labs | NA | NGP0302 |
| 1312 | Anti Maltose Binding Protein | *Mus musculus* | IgG | Dr. Brian Cao, VARI | 5F12 | NA |
| 1311 | Anti Glutathione S-transferase | *Mus musculus* | IgG | Dr. Brian Cao, VARI | 8C1 | NA |
| 1310 | Anti *Bacillus anthracis* Lethal Factor (Negative Control) | *Mus musculus* | IgG | Dr. Brian Cao, VARI | NA | NA |
| 1309 | Anti Blood Group H | *Mus musculus* | IgM | Abcam | 87-N | ab24222 |
| 1306 | Anti Blood Group Lewis b | *Mus musculus* | IgG | Thermo Scientific | 2-25LE | MA1-19346 |
| 1299 | Anti Blood Group A | *Mus musculus* | IgG | Abcam | 9A | ab20131 |
| 1298 | Anti Blood Group B | *Mus musculus* | IgM | Abcam | Z5H-2 | ab24224 |
| 1098 | Anti Mucin 16 | *Mus musculus* | IgG | Novus Biologicals | X306 | NB120-10032 |
| 995 | Anti Bradykinin | *Orytolagus cuniculus* | IgG | Abcam | NA | ab47686 |
| 855 | Anti Mucin 1 | *Mus musculus* | IgG | Neoclone | 614D | NA |
| 822 | Neu5Aca2,3Galb1,4(Fuca1,3)Glc-sp-BSA | NA | Glycoconjugate | Vector Labs | NA | NGP0405 |
| 499 | Anti Blood Group Lewis a | *Mus musculus* | IgG | Abcam | 7LE | ab3967 |
| 342 | Anti-Carcinoembryonic Antigen (CEA) | *Mus musculus* | IgG | USBiological | 2.Q.397 | C1299-94 |
| 194 | Anti Von Willebrand factor | *Orytolagus cuniculus* | Ig | DAKO | NA | A0082 |
| 831 | Anti Mucin 5AC | *Mus musculus* | IgG | AbD Serotec | 45M1 | 1695-0128 |
| 1251 | Anti Mucin 5AC | *Mus musculus* | IgG | Thermo Scientific | 2-11M1 | MA1-35704 |
| 1205 | Anti Mucin 3A | *Mus musculus* | IgG | Lifespan Biosciences | Not Listed | LS-C16658 |
| 1192 | Anti Mucin 3 | *Mus musculus* | IgG | Abcam | M3.1 | ab24068 |
| 1202 | Anti Mucin 2 | *Mus musculus* | IgG | Abcam | 994/152 | ab22712 |
| 830 | Anti Mucin 16 | *Mus musculus* | IgG | Abcam | X325 | ab10033 |
| 1093 | Anti Mucin 1 | *Mus musculus* | IgG | GeneTex | CM1 | GTX10114 |
| 370 | Anti Insulin-like Growth Factor 1 | *Capra hircus* | IgG | R&D Systems | NA | AF-291-NA |
| 1249 | Anti Human Milk Fat Globule 1 | *Mus musculus* | IgG | Thermo Scientific | EDM45 | MS-512-P1 |
| 1293 | Anti HA Tag (Negative Control) | *Mus musculus* | IgG | Dr. Brian Cao, VARI | NA | NA |
| 793 | Anti Endorepellin | *Capra hircus* | IgG | R&D Systems | NA | AF2364 |
| 739 | Anti CEACAM6 | *Mus musculus* | IgG | Santa Cruz | By114 | sc-20059 |
| 1303 | Anti Cancer Antigen 19.9 | *Mus musculus* | IgM | Abcam | 121SLE | ab3982 |
| 1295 | Anti Cancer Antigen 19.9 | *Mus musculus* | IgG | USBiological | 9L426 | C0075-03A |
| 955 | Anti Bradykinin | *Orytolagus cuniculus* | Ig | AbD Serotec | NA | 0100-0443 |
| 1279 | Anti *Bacillus anthracis* Protective Antigen (Negative Control) | *Mus musculus* | IgG | Dr. Brian Cao, VARI | 10F5 | NA |
